# *Juniperus phoenicea* L. Essential Oil from Ain El Orak (Algeria): Chemical Analysis by GC/MS, In Vitro Antioxidant and In Vivo/In Silico Gastroprotective and Hepatoprotective Effects

**DOI:** 10.3390/foods15101667

**Published:** 2026-05-11

**Authors:** Meriem Medjekane, Yacine Nait Bachir, Zohra Douaa Benyahlou, Fawzia Nemar, Housseyn Medjahed, Safia Ali Haimoud, Meryem Sadoud, Hiba Naas, Assia Nehari, Messouda Mansouri, Chaima Mimouni, Abdelkader Chouaih, Roberta Foligni

**Affiliations:** 1 Institute of Life and Natural Sciences, Nour Bachir University Center of El-Bayadh, El-Bayadh 32000, Algeria; h.medjahed@cu-elbayadh.dz (H.M.); mansourimessouda4@gmail.com (M.M.); chaimamimouni05@gmail.com (C.M.); 2 Laboratory of Experimental Pathological Research, Department of Nutrition and Food Sciences, Hassiba Benbouali University of Chlef, Ouled Fares, Chlef 02010, Algeria; f.nemar@univ-chlef.dz (F.N.); s.alihaimoud@univ-chlef.dz (S.A.H.); 3 Department of Biology, Faculty of Natural and Life Sciences, University Saad Dahlab–Blida 1, Blida 09000, Algeria; phd.nait.bachir.yacine@gmail.com; 4 Laboratory of Technology and Solid Properties (LTPS), Faculty of Sciences and Technology, Abdelhamid Ibn Badis University of Mostaganem, Mostaganem 27000, Algeria; benyahlou.zohra.douaa@gmail.com (Z.D.B.); achouaih@gmail.com (A.C.); 5 Laboratory of Beneficial Microorganisms, Functional Food and Health, Department of Food Science, Faculty of Nature and Life Science, Abdelhamid Ibn Badis University, Mostaganem 27000, Algeria; ms.sadoud@univ-chlef.dz; 6 Department of Nutritional Sciences, Faculty of Life and Natural Sciences, University of Relizane, Relizane 48000, Algeria; hiba.naas@univ-relizane.dz; 7 Laboratory of Natural Bio-Resources, Department of Nutrition and Food Sciences, Faculty of Life and Natural Sciences, Hassiba Benbouali University of Chlef, Ouled Fares, Chlef 02010, Algeria; a.nehari@univ-chlef.dz; 8 Department of Human Sciences and Promoting of the Quality of Life, San Raffaele University Rome, Via Val Cannuta 247, 00166 Rome, Italy

**Keywords:** gastro-protective, hepato-protective, α-terpinolene, *Juniperus phoenicea*, essential oil

## Abstract

*Juniperus phoenicea* L. is a popular plant in alternative medicine, particularly in the steppe and highland regions of western Algeria. The present study focuses on characterizing the essential oil of *Juniperus phoenicea* growing spontaneously in the Ain El Orak region of El Bayadh province, where it is a valuable resource. The essential oil yield obtained by hydrodistillation was 0.98%, and its characterization by GC-MS revealed 46 compounds, predominantly α-Terpinolene at 21.29%, Limonene at 14.68%, Terpinene 4-ol at 12.04%, β-Myrcene at 9.93%, and β-Pinene at 7.31%. The study of the anti-radical activity against DPPH showed an IC50 value of approximately 0.23 mg/mL. The evaluation of the anti-ulcer property on experimentally induced ulcers in mice through oral administration of ethanol demonstrated excellent protection of the gastric mucosa, with 48.07%, 54.87%, and 81.92% protection for doses of 50, 100, and 200 mg/kg, respectively, comparable to omeprazole at 72.40%. The hepatoprotective activity against toxicity induced by intraperitoneal injection of a 250 mg/kg dose of paracetamol in mice showed a protective effect expressed by the decrease in serum levels of AST (260.33 ± 9.69 IU/L) and ALT (56.22 ± 9.63 IU/L) to values comparable to the those of the physiological group, especially for the 300 mg/kg dose of the essential oil of *J. phoenicea*.

## 1. Introduction

Algeria, a country renowned for its diverse landscapes and climates, is rich in aromatic and medicinal plants. These plants have been utilized in various fields, including pharmacy, perfumery, cosmetics, and food, due to their therapeutic, organoleptic, and aromatic properties. Traditional medicine is deeply rooted in Algerian society, with numerous plants and their extracts employed in traditional therapies. The use of these plants extends beyond benign ailments, encompassing chronic diseases as well [1]. Various forms of administration, such as infusions and topical applications, have been preserved and passed down through generations.

Among these spontaneous plants, *Juniperus phoenicea* L. (commonly known as the Phoenician juniper or juniper tree) is considered an important medicinal plant widely used in traditional medicine as a diuretic, stimulant, gastric tonic, pulmonary disinfectant, and depurative. A decoction of its leaves and berries is used to treat diarrhoea, rheumatism, and diabetes.

*Juniperus phoenicea* L. is distributed worldwide, but is more prevalent in the western Mediterranean region, particularly in southern Europe (from Portugal to Turkey) and western Asia (especially in the mountains of western Saudi Arabia) [2]. In North Africa, it grows in Algeria, Morocco, Tunisia, and Egypt.

Numerous studies have investigated the molecular composition of various extracts and essential oils from the leaves and berries of *J. phoenicea*, revealing a diverse range of compounds, including diterpenoids, biflavonoids, lignans, phenylpropanoid glycosides, furanone glycosides, and norterpene and sesquiterpene glycosides [3,4]. A recent study [5] reported promising results on the essential oils of this species, indicating cytotoxic effects against breast and colon cancer cells, suggesting its potential as a significant source of natural products with pharmacological interest.

Gastrointestinal disorders represent a major and growing global health concern, affecting a large proportion of the population worldwide. Among them, gastric ulcers, gastritis, and gastroesophageal reflux disease are particularly prevalent, and are associated with significant morbidity and reduced quality of life. It is estimated that peptic ulcer disease alone affects approximately 5–10% of the global population during their lifetime, with complications such as bleeding, perforation, and chronic inflammation remaining clinically relevant [6,7,8,9]. *Helicobacter pylori* infection, prolonged use of non-steroidal anti-inflammatory drugs (NSAIDs), alcohol consumption, stress, and unhealthy dietary habits are among the main contributing factors to gastric mucosal damage [10]. Although proton pump inhibitors and other synthetic drugs are widely used, their long-term administration may be associated with adverse effects, relapse after treatment withdrawal, and the emergence of resistant bacterial strains [11,12]. These limitations highlight the urgent need for safer, more effective, and natural alternative therapeutic strategies. In this context, medicinal plants and their bioactive compounds have gained increasing attention as promising sources of gastroprotective agents, due to their antioxidant, anti-inflammatory, and cytoprotective properties [13,14,15].

Inspired by the widespread use of this species for digestive disorders, the primary objective of this study was to evaluate its protective potential for two organs, using experimental models in mice. Consequently, the essential oil from the leaves of *J. phoenicea* (JPEO) was extracted and its phytochemical and biological properties were characterised through GC-MS analysis. Furthermore, its antioxidant activity, gastroprotective effect against ethanol-induced ulcers, and hepatoprotective effect against paracetamol-induced toxicity were evaluated.

## 2. Materials and Methods

### 2.1. Plant Material and Extraction of Essential Oil

The plant material, consisting of *J. phoenicea* leaves, was collected in March 2023 from Ain El Orak (El Bayadh, Algeria). The plant material was authenticated by botanical experts, based on herbarium comparison at the University of Laghouat (Algeria), where a voucher specimen was deposited and assigned the reference number URPM.Jp.03.23.

After cleaning and drying, the plant material was ground into a powder and stored at room temperature in airtight amber glass jars. The *J. phoenicea* essential oil (JPEO) was obtained in the laboratory using hydrodistillation with a Clevenger-type apparatus. The essential oils were stored in vials at 4 °C, protected from light [16].

### 2.2. Gas Chromatography–Mass Spectrometry (GC-MS) Analysis

The analysis was carried out at the Center for Scientific and Technical Research in Physicochemical Analyses (CRAPC), Algiers. The mass spectrometer (5972 quadrupole) coupled to the gas chromatograph (6890) was manufactured by Hewlett-Packard (Palo Alto, CA, USA) 5972 quadrupole, was coupled to a Hewlett-Packard 6890 gas chromatograph. The components of the essential oils were simultaneously separated on an HP-5MS capillary column (30 m × 0.25 mm i.d., 0.25 µm film thickness) coated with 5% phenyl–95% dimethylpolysiloxane. The operating conditions were described in Cardinali et al. [17]: 250 °C for the split injector temperature and 250 °C for the transfer line. The temperature program for the analysis of essential oils was: 40 °C (for 2 min), then increased to 210 °C at a rate of 2 °C/min, and held constant at 210 °C for 30 min.

The identification of the different constituents was carried out based on their Kovats retention indices [18] on both types of columns, in comparison with the literature. Confirmation was provided using mass spectra by comparison with those of standard compounds in the computerized database [19,20].

### 2.3. In Vitro Study (Estimation of Antioxidant Power by the DPPH Method)

The protocol used is that described by [21]. In tubes, 2.5 mL of JPEO (0.1 mg/mL) and 1 mL of the methanolic DPPH solution (0.3 mM) were added. After vortexing, the tubes were placed in the dark at room temperature, for 30 min. The reading was taken by measuring the absorbance at 517 nm using a SHIMADZU^®^-type UV–Visible spectrophotometer (Kyoto, Japan). The results can be expressed as the percentage of radical scavenging activity (I%), using the following formula:

I% of Anti-radical activity = [(A1 − A2)/A1] × 100

I%: percentage of anti-radical activity.

A1: absorbance of the sample.

A2: absorbance of the negative control.

The negative control consisted of 1 mL of the methanolic DPPH solution and 2.5 mL of methanol. Several concentrations were tested (2.5 mg/mL–20 mg/mL) until the ideal concentration of each extract was obtained. The tests were performed in triplicate.

### 2.4. In Vivo Study

#### 2.4.1. Animals

Adult female mice weighing between 25 and 30 g, provided by the Pasteur Institute of Algiers, were used for experimental purposes. The mice were housed under standard conditions (12 h light/dark cycle, temperature at 25 °C, food and water ad libitum) for at least one week, to acclimate to the new conditions before any experiment. Appropriate anaesthesia and sacrifice procedures were followed to ensure that the animals did not suffer at any stage of the experiments. Ethics Committee Name: Institutional Animal Ethics Committee, University of Chlef. Approval Code (2023): D00L01UN20230001 in the scientific institution code C0810600.

#### 2.4.2. Evaluation of the Gastroprotective Activity of Essential Oil

This test involves acutely inducing ulcers in mice with an ulcerogenic solution that produces distinct or measurable ulcers [22]. The anti-ulcer or gastroprotective activity of a substance can be tested by administering it before or after the ulcerogenic agent and evaluating its effect.

The evaluation of the anti-ulcer activity is done by determining and assessing a severity scale of lesions or the ulcer index. To verify the protective action of JPEO against ethanol-induced ulcers in mice, 6 groups of 6 mice were formed. After 24 h of fasting, groups 1 and 2 received 1 mL of the vehicle and omeprazole (30 mg/kg) orally, respectively. The other groups received 50, 100, and 200 mg/kg of JPEO. One hour later, ulceration was induced by intragastric instillation of 0.2 mL of 90% ethanol [23]. The mice were sacrificed by decapitation under light chloroform anaesthesia 30 min after induction of gastric lesions. Their stomachs were removed, opened along the greater curvature, the contents removed, and the gastric mucosa was gently washed with saline solution (0.9%).

The stomachs were imaged so that lesions could be quantified using ImageJ software (National Institutes of Health, Bethesda, MD, USA). The sum of the area of all lesions for each mouse was calculated and used as an ulcer index. The percentage of ulcer inhibition was determined as follows:

% ulcer inhibition = (Ulcer index Et − Ulcer index treated) × 100/Ulcer index Et

Ulcer index Et: ulcer index of mice treated with ethanol.

Ulcer index treated: ulcer index of mice treated with JPEO or omeprazole and ethanol.

#### 2.4.3. Evaluation of the Hepatoprotective Activity of Essential Oil

Animals were divided into 4 groups of 6 mice. JPEO (100 and 300 mg/kg) was administered to mice orally using a gavage needle, while paracetamol (250 mg/kg) was administered intraperitoneally. Food was removed from the mice one hour before each gavage and was returned one hour later.

The preventive effect of the extracts against paracetamol-induced hepatotoxicity was evaluated after administering JPEO for 7 days. On the 7th day, paracetamol was injected 1 h after administration of the last dose of the extract; 24 h later, the mice were sacrificed by decapitation under light chloroform anaesthesia, after an overnight fast. Blood was collected from the jugular vein for the dosage of hepatic parameters, including transaminases [24].

#### 2.4.4. Histological Study

The glandular parts of the stomachs from the animals in the gastroprotective activity test, as well as the livers of the mice subjected to the hepatoprotective activity test, were recovered after dissection, fixed in 10% formalin for histological study, embedded in paraffin wax, sectioned into 5 µm sagittal sections, and stained with haematoxylin–eosin (H & E). Representative areas were selected for qualitative analysis by light microscopy.

#### 2.4.5. Statistical Analyses

The results are expressed as mean ± standard deviation. Statistical significance was determined using one-way analysis of variance (ANOVA), followed by Tukey’s test for pairwise comparisons. A *p*-value of less than 0.05 was considered statistically significant. The statistical analysis was performed using IBM SPSS Statistics V22.0 software.

### 2.5. In Silico Study

Once the experimental results have been obtained, molecular docking can be used to simulate and predict the interactions between molecules and their biological targets, thus providing a structural perspective on their efficacy. This approach makes it possible to identify the most promising compounds, explore their binding sites and optimise their therapeutic potential. In this study, molecular docking was applied to forty-six (46) compounds extracted from *Juniperus phoenicea* essential oil, identified using CPG-SM and obtained from databases such as PubChem [25]. The aim is to evaluate their potential in three areas of activity: antioxidant, anti-ulcer and hepatoprotective, and thus guide future research towards specific therapeutic applications.

For the in silico evaluation of *Juniperus phoenicea* extracts, validated targets were selected and co-crystallised with reference ligands from the Protein Data Bank (PDB) [26] in order to establish the docking parameters in pharmacologically relevant pockets. For antioxidant activity, xanthine oxidoreductase was used in two complexed states: PDB: 3BDJ [27,28] bound to oxypurinol (an active metabolite of allopurinol) and PDB: 3NVY [29] bound to quercetin; these structures allow the catalytic site to be fixed in the vicinity of the molybdenum centre, which is canonical for ROS generation. For anti-ulcer activity, two complementary mechanisms were considered: inhibition of gastric H^+^/K^+^-ATPase (PDB: 5YLU [30]), co-crystallised with the blockers vonoprazan and SCH28080, the terminal target of acid secretion; and inhibition of *Helicobacter pylori* urease (PDB: 6ZJA [31]), co-crystallised with the imidazole hydroxamate SHA, which chelates the bi-Ni^2+^ centre and precisely defines the active cavity responsible for bacterial acid–base tolerance. For hepatoprotective activity, the following were targeted: PPAR-α (PDB: 5HYK [32]), a key nuclear receptor for lipid metabolism and inflammation, in complex with a reference LBD ligand (ligand identifier 65W), relevant for modelling hepatoprotective agonism, as well as ALK5/TGF-βRI kinase (PDB: 1VJY [33,34]), a central node in fibrogenesis, co-crystallised with a 1,5-naphthyridine inhibitor serving as an ATP pocket template for antifibrotic screening.

The selection of target proteins was based on several structural quality criteria. First, only structures determined by X-ray diffraction (XRD) were selected, as this method is recognized as the most reliable for obtaining high atomic resolution. The selected proteins thus have a crystallographic resolution of less than 2.50 Å, which guarantees high precision of the atomic coordinates (see Table 1) [35,36]. In addition, the selected structures include R-free data, an essential parameter for evaluating the quality and validity of crystallographic models. All selected proteins have an R-free value of less than 0.45 [37], reflecting a good correlation between the experimental data and the generated model.

For the simulation, the forty-six ligands were first optimized using Gaussian software (09 Revision A.1) [38] in order to obtain a stable geometry corresponding to a minimum energy state. The receptors were prepared using Discovery Studio [39], removing water molecules and heteroatoms, then adding polar hydrogen atoms and partial charges according to the Kollman model [40].

Docking simulations were performed using PyRx software (version 1.2) [41], incorporating the AutoDock Vina motor (v1.1.2) [42]. Each ligand was docked individually in the same active region as the co-crystallized ligand, allowing the optimal orientation of each ligand in the active site of the target proteins to be predicted, as well as the corresponding interaction energy. The main interactions (hydrogen bonds, hydrophobic forces, π–π interactions) were visualized and validated using Discovery Studio Visualizer [39].

Docking Validation (RMSD): The quality of the poses was confirmed by the re-anchoring of the native ligand of target PDB: 3BDJ with an RMSD < 2 Å, thus ensuring the reliability of the protocol.

## 3. Results and Discussion

### 3.1. Yield and Composition of Essential Oil

The essential oil extracted from the leaves of *J. Phoenicea* from Ain El Orak exhibited a pale yellow colour and yielded 0.98%, based on the dry weight of the sample. Table 2 presents the chemical composition of JPEO as determined by GC-MS.

The analysis revealed 46 compounds, accounting for 96.37% of the essential oil. The major constituents were predominantly α-Terpinolene at 21.29%, Limonene at 14.68%, Terpinene 4-ol at 12.04%, β-Myrcene at 9.93%, and β-Pinene at 7.31%. Other components such as γ-Terpinene, Sabinene, β-Pinene, α-Pinene, and Borneol were present in lower proportions (between 7 and 3%), while the remaining constituents were found in equal or lower quantities.

The composition of JPEO from El Bayadh was notably different, due to the presence of 11 new compounds, particularly the major compound α-Terpinolene, as well as Terpinene 4-ol, p-mentha-3,8-diene, 2-Nonanone, 2-Undecanone, Pyronene, Benzyl isovalerate, Geranyl acetone, Valencene, Benzyl Benzoate, and Hexahydrofarnesyl acetone.

Few studies have been conducted in Algeria on JPEO. Menaceur and colleagues (2013) [43], in a study on a sample from Bouira, reported 65 compounds, primarily α-pinene at 34.4%, followed by terpinenyl acetate (7.2%), δ-3-carene (5.9%), δ-cadinene (5.8%), and cadina-1-4-diene (4.9%). Dob et al. (2008) [44] identified over 130 constituents, including α-pinene (40.2%), α-phellandrene (14.7%), and elemol (3.9%) in a juniper sample from El Djelfa, which shares similar semi-arid climatic conditions with El Bayadh, the collection area of the sample of the present study.

The essential oil extracted from the leaves of wild juniper in the Green Mountain (al djabal al akhdar) in northern Libya contained 16 compounds, primarily characterised by α-terpinyl acetate (13.21%), α-pinene (12.63%), and germacene D (11.49%) [45]. According to Jemaï Ben Ali and colleagues (2015) [46], the yield of JPEO from Gafsa (Tunisia) was 0.96%, and it was composed mainly of α-pinene (20.24%), naphthalene (6.65%), linalool (3.72%), myrcene (3.25%), p-cymène (2.00%), and bicyclosesphellandrène (1.77%).

Various factors can play a crucial role in chemical composition, including environmental factors such as geography, harvest season (day length and temperature), and the nutritional status of the plant [47].

A comparative study between Moroccan and Tunisian JPEO [48] mentioned 13 compounds common to both essential oils, as well as numerous other differences, including the number of compounds (31 for Moroccan JPEO and 45 for Tunisian JPEO), the content of the major compound α-pinene (higher for Moroccan JPEO), and the levels of δ-3-carene and limonene (higher for Tunisian JPEO), reflecting the effect of geographic distribution on the composition of essential oils once again.

### 3.2. In Vitro Antioxidant Activity

The DPPH radical scavenging activity of JPEO was evaluated. The results indicate a significant antioxidant activity, with an IC50 of 0.23 mg/mL.

According to Ghouti et al. (2018) [49], JPEO from southwestern Algeria exhibited an IC50 of 0.76 mg/mL, while Loizzo et al. (2007) [50] reported an IC50 of 0.0742 mg/mL. The essential oil from the leaves of *J. phoenicea* has shown a higher antioxidant effect compared to its berries, as reported by Mansour et al. (2023) [5].

It has been suggested that terpinolene, the major compound in oil, possesses the ability to scavenge reactive species (de Christo Scherer et al., 2019) [51]. Monoterpene hydrocarbons and oxygenated monoterpenes may be the primary contributors to this activity [52].

The antioxidant effect of essential oils can occur through various mechanisms, including the elimination of free radicals, prevention of chain initiation, termination of peroxides, and reduction of agent activity. This may result from synergistic, antagonistic, and/or additive effects of the different components [53].

While synthetic antioxidants such as BHA and BHT are highly effective, they may exhibit side effects, and even toxicity [54]. To mitigate the side effects and toxicity of synthetic products, scientists are compelled to turn to phytotherapy.

### 3.3. Evaluation of the Gastroprotective Activity of Essential Oil

**Figure 1:**
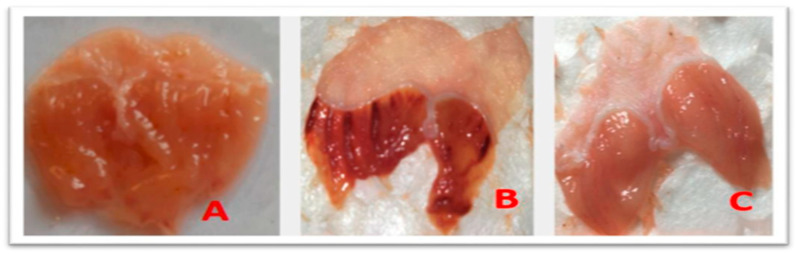
Photographs of macroscopic observations of stomachs. (**A**) Healthy stomach; (**B**) ulcerated stomach; (**C**) stomach pretreated with omeprazole.

**Figure 2:**
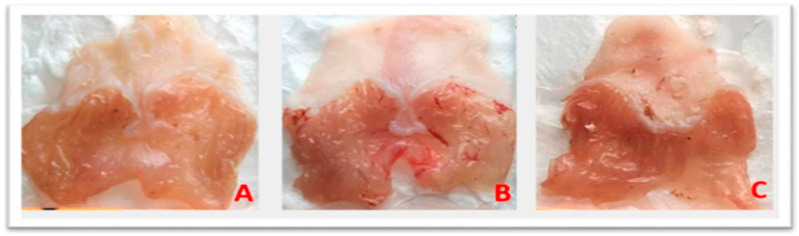
Photographs of macroscopic observations of JPEO-pretreated stomachs. (**A**) 50 mg/kg, (**B**) 100 mg/kg, (**C**) 200 mg/kg.

The macroscopic study of the anti-ulcer or gastroprotective activity of the juniper essential oil was evaluated by oral administration to mice prior to an ulcerogenic agent, ethanol, which induces measurable ulcers. Gavage with 0.2 mL of 90% ethanol in animals induced extensive haemorrhagic gastric ulcers (Figure 1B) and a necrotic appearance, in contrast to the stomachs of the healthy group (Figure 1A), which did not exhibit any of these characteristics, with an ulcer index of 55.31% ± 1.22 (Table 3). Pretreatment with omeprazole, a proton pump inhibitor that reduces gastric acid secretion, revealed an ulcer index of 15.27 ± 0.33 (Figure 1C, Table 3), corresponding to a 72.40% inhibition rate of gastric lesions. Pretreatment with JPEO inhibited ethanol-induced ulcers (Figure 2A–C) in a dose-dependent manner, with ulcer indices of 28.72 ± 1.74, 24.96 ± 1.38, and 10 ± 0.00 for doses of 50, 100, and 200 mg/kg, respectively. The percentages of inhibition of gastric lesions were then 48.07%, 54.87%, and 81.92% for doses of 50, 100, and 200 mg/kg, respectively (Table 3). These results indicate that the essential oil of the present study exerts a gastroprotective effect. The best ulcer inhibition rates appeared at the highest doses, demonstrating a dose-dependent effect. In general, treatment of ulcers with juniper or omeprazole provides good protection of the stomach and reduces ulcer scores.

**Figure 3:**
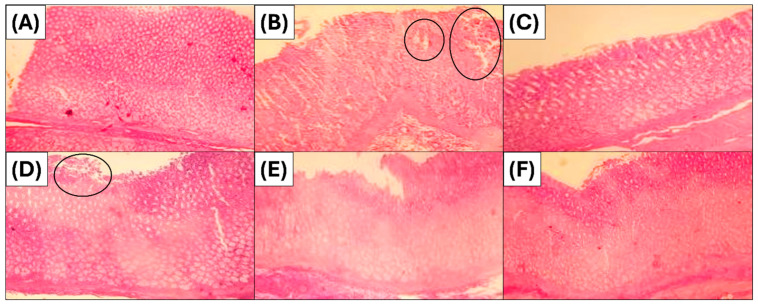
Microphotographs representing the histological structures of the stomachs. (**A**): Group 1 (physiological stomach treated with physiological saline solution). (**B**): Group 2 (negative control receiving ethanol only). (**C**): Group 3 (positive control treated with Omeprazole 20 mg/kg). (**D**): Group 4 (treated with JPEO 50 mg/kg). (**E**): Group 5 (treated with JPEO 100 mg/kg). (**F**): Group 6 (treated with JPEO 200 mg/kg).

To confirm the results obtained in the macroscopic evaluation, a histopathological study was conducted on the stomachs. This study provided a better assessment of the gastroprotective activity of the essential oil. The results are presented in Figure 3. Figure 3A shows a microphotograph of a physiological stomach with a healthy structure of the mucosa and submucosa, displaying a normal epithelial structure. Ulcerations, epithelial desquamation, and haemorrhages were observed in group 2, the negative control (Figure 3B). Omeprazole provided excellent protection of the gastric tissue (Figure 3C), as was observed in a normal tissue structure with no histological changes. The histological study also showed a good gastroprotective effect of JPEO at doses of 200 mg/kg (Figure 3F) and 100 mg/kg (Figure 3E). However, at a dose of 50 mg/kg (Figure 3D), this essential oil was not effective, as significant epithelial desquamation and ulcerations were observed in this microphotograph.

It should be noted that the ethanol-induced ulcer model is widely used in in vivo experiments to evaluate the gastroprotective activity of various agents from natural resources. Experimentally, intragastric administration of ethanol has been shown to produce elongated haemorrhagic bands, extensive submucosal oedema, mucosal friability, infiltration of inflammatory cells, and loss of epithelial cells. Moreover, alcohol has a triple ulcer-inducing action: it erodes the mucosa, causes congestion, and induces cell necrosis [55]. Many medicinal plants have in their chemical composition flavonoids, triterpenoids, and tannins, which protect the gastric mucosa by inducing gastroprotective mechanisms or acting as natural antioxidants [56]. Results were in agreement with the work of a Tunisian team [46], who demonstrated the potent anti-ulcer effect of *Juniperus phoenicea* essential oil linked to antioxidant activity by reducing MDA levels and increasing the defensive factors of the gastric mucosa GSH, GST, GPx, CAT, and SOD.

### 3.4. Evaluation of the Hepatoprotective Activity of Essential Oil

The hepatoprotective activity of JPEO was evaluated using mice as an experimental model. Hepatotoxicity was induced by intraperitoneal injection of a 250 mg/kg dose of paracetamol (Table 4).

Serum levels of ALT (103.34 ± 18.96 IU/L) and AST (380.36 ± 22.33) in the paracetamol-treated group (250 mg/kg) were significantly higher compared to the vehicle group that received physiological saline, where ALT and AST were (58.66 ± 8.33 IU/L) and (234 ± 12.24), respectively. JPEO at 300 mg/kg restored ALT (56.22 ± 9.63) and AST (260.33 ± 9.69) levels to physiological levels comparable to those recorded in the vehicle group. These parameters appeared slightly higher for the 100 mg/kg dose (ALT at 62.38 ± 10.36 and AST at 300.12 ± 25.6).

**Figure 4:**
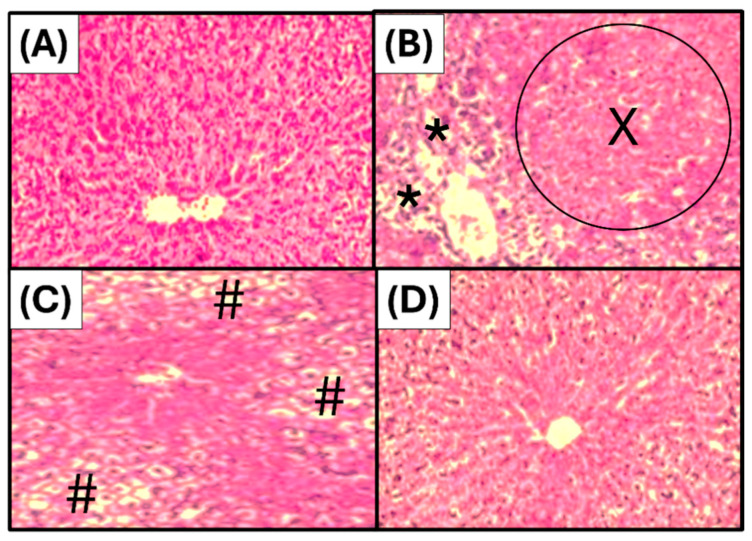
Microphotographs representing the histological structures of the liver. (**A**): Group 1 (physiological liver treated with the vehicle). (**B**): Group 2 (negative control receiving paracetamol only). (**C**): Group 3 (treated with JPEO 100 mg/kg). (**D**): Group 4 (treated with JPEO 300 mg/kg). (*): Inflammatory infiltration in the centrilobular area. (X): Presence of large necrotic areas in the hepatic parenchyma. (#): Ballooning degeneration of hepatocytes.

The microphotograph shows a physiological structure of hepatic tissue, with the liver organized into lobules composed of hepatocytes arranged radially around a central vein (Figure 4A). The administration of paracetamol induced histological alterations, mainly the infiltration of inflammatory cells, congested central vein, sinusoidal dilation, dissociation of hepatic tissue, necrotic cells, and cell shadowing (Figure 4B). The administration of JPEO at 100 mg/kg showed a slight reduction in paracetamol-induced hepatitis. Mild periportal hepatitis and hepatic tissue infiltrated by inflammatory cells, along with ballooning degeneration of hepatocytes, was observed (Figure 4C). On the other hand, a dose of 300 mg/kg of JP EO demonstrated an excellent hepatoprotective effect, with the hepatic tissue appearing nearly normal, hepatocytes being normal, and only slight periportal inflammatory cell infiltration (Figure 4D).

The experimental period lasted 7 days, which is widely considered sufficient in the literature to evaluate the potential hepatoprotective effect of natural substances [57,58,59]. The experimental model based on the use of a single dose of paracetamol is sufficient to induce hepatotoxicity [60]. The intraperitoneal route was adopted because the effect it induces is faster than the intragastric route. However, it is less effective than the intravenous route, which unfortunately is difficult to perform in mice.

Other models of hepatotoxicity based on the use of other substances exist, such as aluminium, which requires multiple doses and a longer experimental duration [61].

The effect of *J. phoenicea* extracts on biomarkers (ALT and AST) was tested, as any alteration in the levels of these parameters is considered a sign of toxicity. ALT is an enzyme specific to the liver, found in the cytosol of hepatocytes, and is strongly released into the blood during hepatocyte lysis. AST, on the other hand, is found in the mitochondria of hepatocytes, kidneys, brain, and skeletal muscle [60]. It is excessively released into the bloodstream following structural damage to the liver.

Our results corroborate the study by Ali and colleagues (1995) [62], who evaluated the hepatoprotective effect of Egyptian juniper against CCL4 (another experimental model) by studying enzymatic biomarkers and histological sections of the liver.

### 3.5. In Silico Study

The results of molecular docking for antioxidant activity showed a wide variation in the affinity of different molecules extracted from JPEO for xanthine oxidoreductase (XO).

ΔG values ranged from −7.6 kcal/mol to −9.1 kcal/mol, suggesting diverse interactions with the enzyme’s active site. Some molecules, such as Benzyl Benzoate (−9.1 kcal/mol), bind strongly, while compounds such as Terpinene 4-ol and α-Pinene have a weaker affinity, −6.3 kcal/mol and −5.7 kcal/mol, respectively. Compared to reference inhibitors, such as Oxypurinol (for 3BDJ, −7.6 kcal/mol) and Quercetin (for 3NVY, −9.2 kcal/mol), several molecules in the plant have comparable or even higher affinity, suggesting that they may exert a similar or potentially complementary antioxidant effect. Furthermore, it is important to note that even molecules with lower affinities may contribute to the overall antioxidant effect of the plant, through a synergistic effect. The interactions of several molecules, even with weaker affinities, may enhance the overall efficacy of the essential oil, creating a more robust antioxidant response.

**Figure 5:**
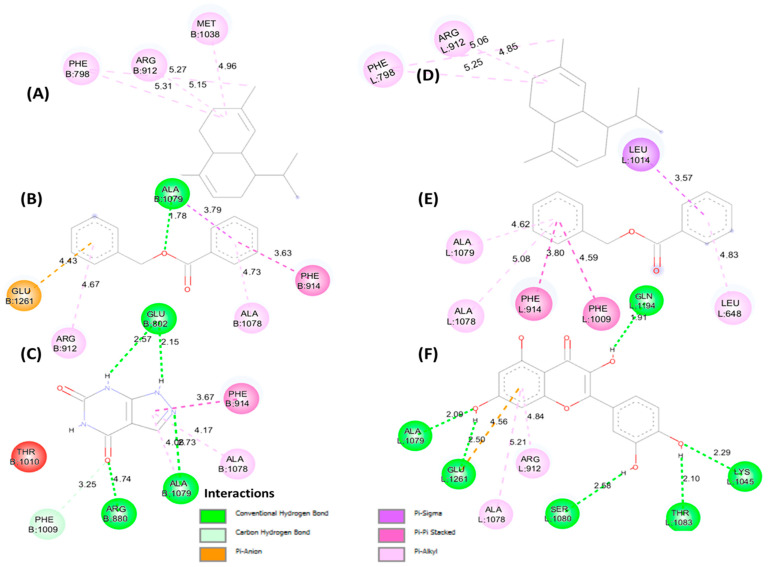
2D representation of molecular interactions for the antioxidant activity: (**A**) complex of xanthine oxidoreductase (3BDJ) with benzyl benzoate, (**B**) complex with α-cadinene, and (**C**) complex with the reference ligand (oxypurinol); (**D**) complex of xanthine oxidoreductase (3NVY) with benzyl benzoate, (**E**) complex with α-cadinene, and (**F**) complex with the reference ligand (quercetin).

**Figure 6:**
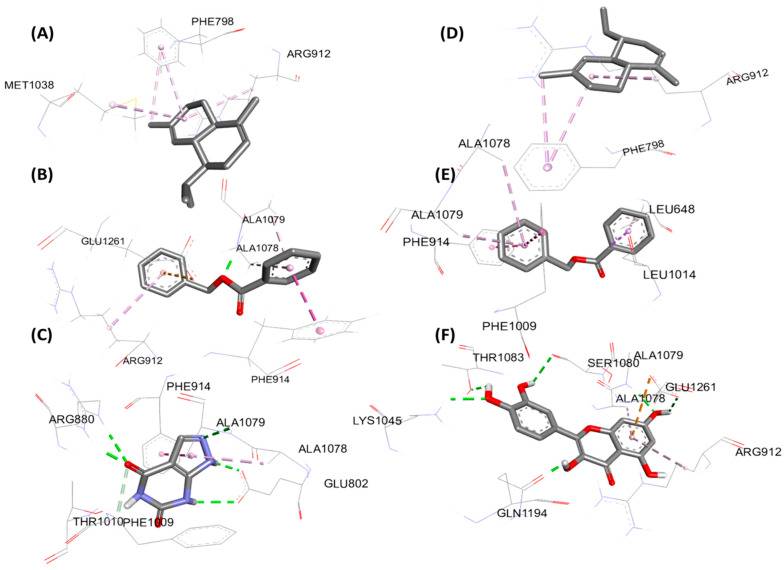
3D representation of molecular interactions for the antioxidant activity: (**A**) complex of xanthine oxidoreductase (3BDJ) with benzyl benzoate, (**B**) complex with α-cadinene, and (**C**) complex with the reference ligand (oxypurinol); (**D**) complex of xanthine oxidoreductase (3NVY) with benzyl benzoate, (**E**) complex with α-cadinene, and (**F**) complex with the reference ligand (quercetin).

Figure 5 (2D) and Figure 6 (3D) illustrate the key interactions in the xanthine oxidoreductase pocket. Protein 3BDJ: (A) benzyl benzoate, (B) α-cadinene, (C) reference ligand oxypurinol. Protein 3NVY: (D) benzyl benzoate, (E) α-cadinene, (F) reference ligand quercetin. The diagrams highlight the dominant hydrophobic contacts, the H bonds of the reference ligand, and the differential occupation of the aromatic sub-pockets.

The results of molecular docking performed on the H^+^/K^+^-ATPase (5YLU) and *Helicobacter pylori* urease (6ZJA) targets showed a wide variability in affinities between JPEO compounds. For H^+^/K^+^-ATPase, ΔG values ranged from approximately −6.1 to −8.6 kcal/mol, indicating interactions of different strengths, depending on the structure of the molecules. The compounds α-copaene (−8.6 kcal/mol), valencene (−7.9 kcal/mol) and α-calacorene (−7.8 kcal/mol) stood out for their strongest affinities, reflecting a high capacity to bind to the active site of the proton pump and potentially block gastric acid secretion. However, molecules that are lighter and less functionalized, such as 2-nonanone (−5.1 kcal/mol), β-myrcene (−6.1 kcal/mol) and p-cymene (−6.6 kcal/mol), have weaker affinities, suggesting less favourable interaction. In comparison, the reference ligand Vanoprazan has a binding energy of −8.4 kcal/mol, which is close to the best molecules in the plant. Vanoprazan’s slightly higher affinity can be explained by its rigid aromatic structure and the presence of nitrogenous and heterocyclic groups, designed to anchor deeply into the catalytic cavity and stabilize the bond through optimal hydrogen and π–π interactions.

For urease (6ZJA), affinity values range from −4.1 to −6.6 kcal/mol, reflecting interactions that are generally lower than those observed for H^+^/K^+^-ATPase. The best results were obtained with α-copaene (−6.6 kcal/mol), α-calacorene (−6.1 kcal/mol) and α-cadinol (−6.1 kcal/mol), indicating a moderate ability to bind to the enzyme’s binuclear nickel site. The reference inhibitor SCH28080 shows higher affinity (−7.3 kcal/mol), due to its aromatic structure and electron-donating atoms, which are able to effectively coordinate the metal ions of the active site, resulting in more specific inhibition. The compounds in JPEO, which are mainly hydrocarbon-based and low in polarity, bind less strongly, due to the lack of functional groups suitable for metal coordination.

Globally, although some molecules in the essential oil, notably α-copaene, valencene, and α-calacorene, have affinities comparable to the reference inhibitors, other less active compounds likely contribute to the overall anti-ulcer effect, through synergy. This synergy between highly and weakly affine molecules would allow for more extensive inhibition of enzymatic pathways, explaining the good biological activity observed experimentally for the crude extract of *Juniperus phoenicea*.

**Figure 7:**
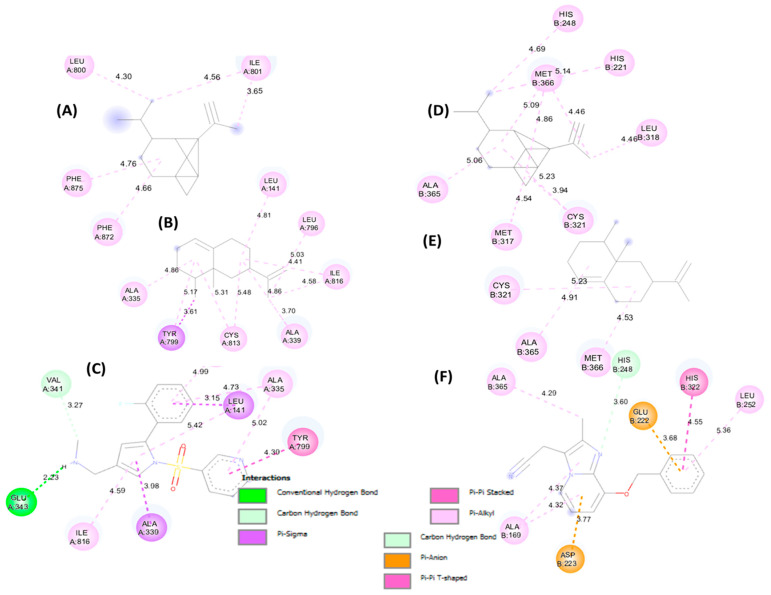
2D representation of molecular interactions for the anti-ulcer activity: (**A**) complex of protein PDB: 5YLU with α-copaene, (**B**) complex with valencene, and (**C**) complex with the reference ligand (Vanoprazan); (**D**) complex of 6ZJA with α-copaene, (**E**) complex with valencene, and (**F**) complex with the reference ligand (SCH22080).

**Figure 8:**
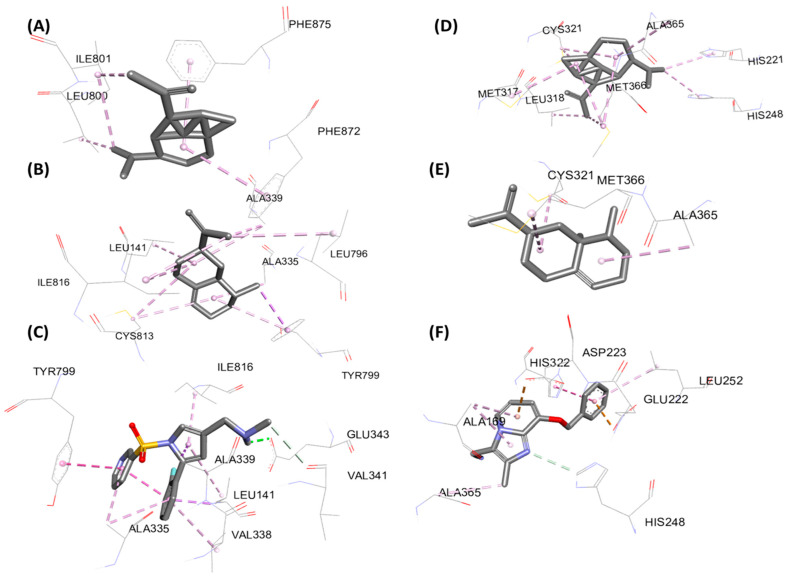
3D representation of molecular interactions for the anti-ulcer activity: (**A**) complex of protein PDB: 5YLU with α-copaene, (**B**) complex with valencene, and (**C**) complex with the reference ligand (Vanoprazan); (**D**) complex of 6ZJA with α-copaene, (**E**) complex with valencene, and (**F**) complex with the reference ligand (SCH22080).

Figure 7 (2D) and Figure 8 (3D) illustrate key contacts within gastric H^+^/K^+^-ATPase (5YLU) and H. pylori urease (6ZJA). Panels (A–C) show 5YLU with the main plant ligands and the reference inhibitor; panels (D–F) show 6ZJA in the same way. These views highlight pocket occupancy, hydrogen bonds of the references, and hydrophobic stacking of sesquiterpenes.

For hepatoprotective activity, *Juniperus phoenicea* compounds show strong profiles on PPAR-α (5HYK) and acceptable profiles on ALK5/TGF-βRI (1VJY). On 5HYK, valencene (−9.2 kcal/mol), α-cubebene (−9.1) and α-calacorene (−9.1) significantly exceed the reference ligand 65W (−6.8), consistent with a highly lipophilic LBD pocket favourable to voluminous sesquiterpenes. The lowest are 2-nonanone (−5.2) and other short aliphatic compounds. On 1VJY, α-copaene (−8.6), aromadendrene (−8.3) and benzyl benzoate (−8.3) show good affinities, but are lower than naphthyridine (−9.3), which is optimised for the ATP pocket, due to its aromatic flatness and donor/acceptor heteroatoms. The weakest are tricyclene (−4.9), α-pinene (−5.0) and borneol (−5.0). The combination of all these compounds suggests an expected synergistic effect: a few high-affinity ligands drive the effect, while medium-affinity ligands stabilise and extend the binding site area, which reinforces the global response of the extract.

**Figure 9:**
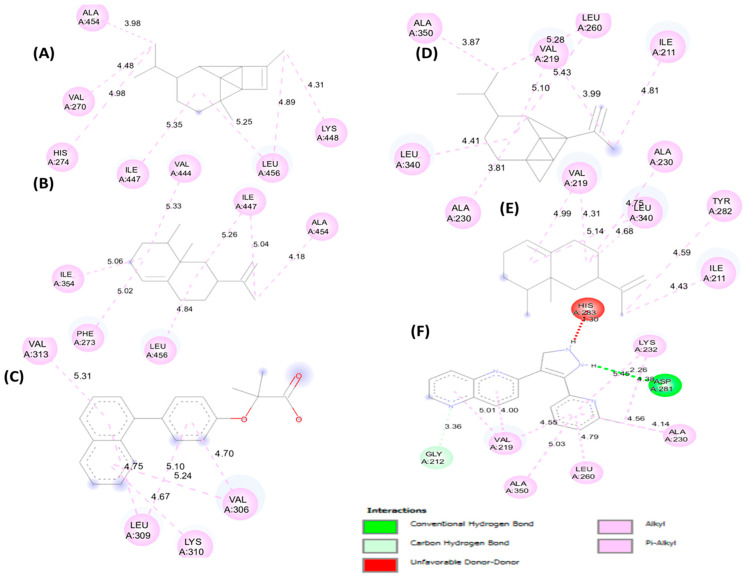
2D representation of molecular interactions for the hepatoprotective activity: (**A**) complex of protein PDB: 5HYK with α-copaene, (**B**) complex with valencene, and (**C**) complex with the reference ligand (65W); (**D**) complex of 1VJY with α-copaene, (**E**) complex with valencene, and (**F**) complex with the reference ligand (Naphthyridine).

**Figure 10:**
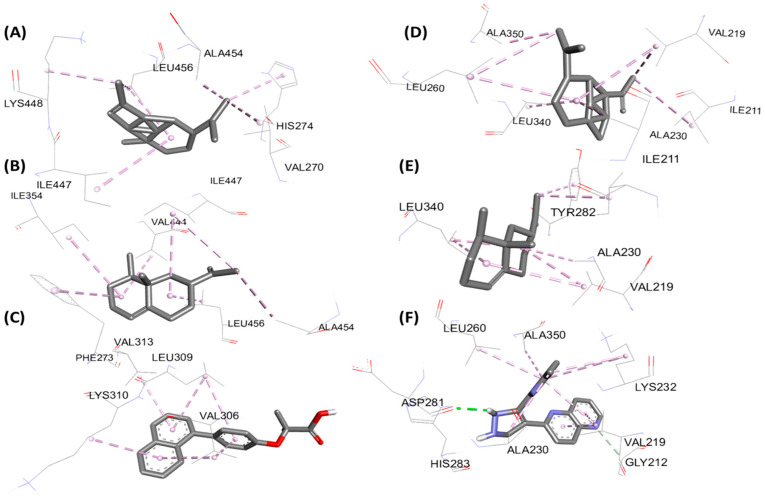
3D representation of molecular interactions for the hepatoprotective activity: (**A**) complex of protein PDB: 5HYK with α-copaene, (**B**) complex with valencene, and (**C**) complex with the reference ligand (65W); (**D**) complex of 1VJY with α-copaene, (**E**) complex with valencene, and (**F**) complex with the reference ligand (Naphthyridine).

Figure 9 (2D) and Figure 10 (3D) show the key contacts for PPAR-α (5HYK) and ALK5/TGF-βRI (1VJY). Parts (A–C) represent 5HYK with the main plant ligands and the reference; the other parts (D–F) represent 1VJY in the same way. The views highlight the hydrophobic fit in the PPAR-α LBD and the anchoring of the ATP pocket for ALK5.

Structure–activity relationship (SAR):

Affinity profiles can be explained by simple rules of steric complementarity and interaction chemistry, specific to each target. On XO (3BDJ, 3NVY), the most effective ligands combine a flat aromatic surface with polar docking possibilities. Benzyl benzoate takes advantage of a large π surface and an orientable ester, which maximises hydrophobic contacts in the XO cavity and explains its high scores (up to −9.1 kcal/mol). Conversely, non-polar and compact monoterpenes (α-pinene, tricyclene) lack H donors/acceptors and show lower ΔG values. The reference quercetin remains the best on 3NVY because its multiple heteroatoms stabilise a network of hydrogen interactions in the proximity of the catalytic centre, a motif absent from the majority of terpenes in the essential oil.

For anti-ulcer activity, H^+^/K^+^-ATPase (5YLU) favours large, hydrophobic ligands capable of occupying the luminal pocket: bicyclic sesquiterpenes (α-copaene, valencene) optimise cavity filling and outperform linear monoterpenes. On urease (6ZJA), the coordination chemistry of the bi-Ni^2+^ centre requires finely positioned heteroatoms; hydrocarbon terpenes are limited to van der Waals interactions and remain in the background, while alcohol sesquiterpenes (α-cadinol) gain modestly from an OH anchor. The references outperform or equal the best compounds for reasons of chemical design: vonoprazan has rigid heteroaromaticity and H-bond sites optimised for the pump, while SCH28080 offers geometry and heteroatoms suited to the enzyme cavity.

In hepatoprotection, PPAR-α (5HYK) has a highly lipophilic LBD: large sesquiterpenes like valencene, α-cubebene, and α-calacorene maximize contact surface and surpass the reference ligand 65W, while smaller aliphatic ketones such as 2-nonanone interact less extensively. In contrast, ALK5 kinase (1VJY) favours a more constrained and polarizable ATP pocket: the reference naphthyridine dominates, due to its aromatic flatness and directional heteroatoms; sesquiterpene hydrocarbons like α-copaene and aromadendrene bind through hydrophobic interactions, but remain weaker because they lack H-bonding or ionic docking sites.

## 4. Conclusions

This study provides a comprehensive investigation of the essential oil of *Juniperus phoenicea* L. collected from Ain El Orak (El Bayadh, Algeria), integrating chemical characterization with in vitro, in vivo, and in silico evaluations of its biological activities. GC–MS analysis revealed a distinctive chemical profile dominated by α-terpinolene, along with other constituents contributing to the specificity of this geographical origin. The essential oil exhibited notable antioxidant activity, which may be attributed to synergistic effects among its monoterpene and sesquiterpene constituents, with significant radical scavenging activity observed at the tested concentration range (2.5–20 mg/mL) and an IC50 value of 0.23 mg/mL. In vivo experiments demonstrated a clear dose-dependent gastroprotective effect against ethanol-induced gastric lesions, with inhibition rates of 48.07%, 54.87%, and 81.92% at 50, 100, and 200 mg/kg, respectively, the highest dose showing greater protection than omeprazole (72.40%). Histopathological analysis confirmed the preservation of gastric mucosal integrity, particularly at 100 and 200 mg/kg. Furthermore, the essential oil showed significant hepatoprotective activity against paracetamol-induced toxicity. This was evidenced by a marked normalization of serum ALT and AST levels, especially at 300 mg/kg, where values approached those of the physiological control group, alongside a near-complete restoration of liver histoarchitecture. Molecular docking studies provided mechanistic insights into these biological effects, highlighting strong interactions of major compounds with key therapeutic targets including H^+^/K^+^-ATPase, *Helicobacter pylori* urease, xanthine oxidoreductase, and PPAR-α. These findings support the hypothesis that the observed pharmacological activities result from a multi-target and synergistic action of the essential oil constituents. Overall, this work highlights *Juniperus phoenicea* essential oil as a promising natural source of bioactive compounds with potential applications in the management of oxidative stress-related gastric and hepatic disorders. Future studies should focus on detailed toxicity profiling and pharmacokinetic and bioavailability assessments, as well as mechanistic and clinical investigations, to further validate its therapeutic potential and support its development as a phytopharmaceutical agent.

## Figures and Tables

**Table 1:** The names, codes, resolution R-free values of the proteins used, and the box dimensions for each active site.

Activity/Target	Code Id	Name of the Protein	Resolution (Å)	R-Value Free	Dimension Box
Antioxidant	3BDJ	Crystal Structure of Bovine Milk Xanthine Dehydrogenase with a Covalently Bound Oxipurinol Inhibitor	2	0.224	x = 94, y = −5, z = 108
	3NVY	Crystal Structure of Bovine Xanthine Oxidase in Complex with Quercetin	2	0.237	x = 88, y = 8, z = 16
Anti-ulcer	5YLU	Crystal structure of the gastric proton pump complexed with vonoprazan	2.8	0.288	x = 49, y = −16, z = −4
	6ZJA	*Helicobacter pylori* urease with inhibitor bound in the active site	2.0	0.224	x = 224, y = 253, z = 199
Hepatoprotective	5HYK	Crystal structure of the complex PPARalpha/AL26-29	1.83	0.262	x = 9, y = 34, z = 19
	1VJT	Crystal structure of Alpha-glucosidase (TM0752) from Thermotoga maritima at 2.50 A resolution	2.50	0.256	x = 16, y = 67, z = 4

**Table 2:** Chemical composition of *Juniperus phoenicea* L. essential oil determined by GC-MS.

N°	Compounds	%	RI (*)
1	Tricyclene	0.82	925
2	α-Thujene	0.91	929
3	α-Pinene	4.91	937
4	Sabinene	7.03	963
5	β-Pinene	5.02	974
6	β-Myrcene	3.37	990
7	α-Phellandrene	1.03	1004
8	δ-3-Carene	t (b)	1013
9	p-Cymene	1.2	1023
10	Limonene	14.68	1023
11	(Z)-β-Ocimene	0.39	1035
12	(E)-β-Ocimene	0.02	1041
13	γ-Terpinene	7.04	1059
14	p-mentha-3,8-diene	t	1068
15	2-Nonanone	0.32	1074
16	α-Terpinolene	21.29	1088
17	Borneol	4.22	1143
18	Terpinen-4-ol	12.04	1157
19	Bornyl acetate	0.63	1283
20	2-Undecanone	0.71	1289
21	α-Cubebene	0.02	1338
22	γ-Pyronene	0.08	1335
23	Ylangene	0.12	1366
24	α-Copaene	0.3	1370
25	β-Elemene	0.66	1392
26	Benzyl isovalerate	0.03	1394
27	β-Caryophyllene	0.92	1420
28	Aromadendrene	0.02	1440
29	α-Amorphene	0.64	1443
30	Geranyl acetone	0.19	1454
31	α-Humulene	1.31	1458
32	Germacrene-D	t	1479
33	Valencene	0.44	1481
34	α-Muurolene	0.63	1497
35	(E.E)-α-Farnesene	0.97	1506
36	δ-Cadinene	0.21	1521
37	Cadina-1,4-diene	0.36	1533
38	α-Cadinene	0.34	1538
39	α-Calacorene	0.2	1544
40	Nerolidol	0.32	1560
41	Caryophyllene oxide	0.65	1582
42	T-Muurolol	0.73	1647
43	β-Eudesmol	0.45	1653
44	α-Cadinol	0.89	1659
45	Benzyl Benzoate	0.02	1762
46	Hexahydrofarnesyl acetone	0.24	1841
Total identified compounds		96.37	

Compounds listed in order of elution from the HP-5 MS column. RI (*): Retention Index. t (b) = trace, less than 0.05%.

**Table 3:** Effect of JPEO pretreatment on ethanol-induced gastric injury in mice.

N°	Treated Groups	Mean Gastric Lesion Indices (%)	Inhibition of Lesions (%)
Group 1	Physiological saline solution	0	0
Group 2	Ethanol	55.31 ± 1.22	0
Group 3	Omeprazole (20 mg/kg) + Ethanol	15.27 ± 0.33 (*)	72.40 (*)
Group 4	JPEO (50 mg/kg) + Ethanol	28.72 ± 1.74 (*), (**), (****)	48.07 (*), (**), (****)
Group 5	JPEO (100 mg/kg) + Ethanol	24.96 ± 1.38 (*), (**), (***), (****)	54.87 (*), (**), (***), (****)
Group 6	JPEO (200 mg/kg) + Ethanol	10 ± 0.00 (*), (**)	81.92 (*), (**)

(*): Significant difference between group 2 (negative control) and the other groups (3, 4, 5, and 6). (**): Significant difference between group 3 (positive control) and the other groups (4, 5, and 6). (***): Significant difference between group 6 treated with JPEO (200 mg/kg) and the other two groups treated with JPEO, group 4 (50 mg/kg) and group 5 (100 mg/kg). (****): Non-significant difference between group 5 treated with JPEO (100 mg/kg) and group 4 treated with JPEO (50 mg/kg).

**Table 4:** Effect of JPEO on serum transaminase levels in mice intoxicated by paracetamol.

Serum Transaminase Levels	Treated Groups (mg/kg)			
	GN (Group 1)	GP (Group 2)	JPEO 100 (Group 3)	JPEO 300 (Group 4)
ALAT (UI/L)	58.66 ± 8.33	103.34 ± 18.96 (*)	62.38 ± 10.36 (**), (***)	56.22 ± 9.63 (**), (***),(****)
ASAT (UI/L)	234 ± 12.24	380.36 ± 22.33 (#)	300.12 ± 25.6 (#), (###)	260.33 ± 9.69 (##), (###),(####)

GN: Normal group treated with vehicle. GP: Paracetamol group (250 mg/kg). HE 100: Group treated with JPEO (100 mg/kg) and paracetamol (250 mg/kg). HE 300: Group treated with JPEO (300 mg/kg) paracetamol (250 mg/kg). ALAT: alamine aminotransferase. ASAT: Aspartate aminotransferase. (*): Significant difference between group 1 (physiological control) and group 2 (negative control). (**): Non-significant difference between group 1 (physiological control) and the two other groups treated with JPEO (3 and 4). (***): Significant difference between group 2 (negative control) and the other groups treated with JPEO (3 and 4). (****): Significant difference between group 3 treated with JPEO (100 mg/kg) and group 4 treated with JPEO (300 mg/kg). (#): Significant difference between group 1 (physiological control) and the groups 2 (negative control) and 3 treated with JPEO (100 mg/kg). (##): Non-significant difference between group 1 (physiological control) and group 4 treated with JPEO (300 mg/kg). (###): Significant difference between group 2 (negative control) and the other groups treated with JPEO (3 and 4). (####): Significant difference between group 3 treated with JPEO (100 mg/kg) and group 4 treated with JPEO (300 mg/kg).

## Data Availability

The original contributions presented in this study are included in the article. Further inquiries can be directed to the corresponding authors.
